# A Novel Parallel Multi-Scale Attention Residual Network for the Fault Diagnosis of a Train Transmission System

**DOI:** 10.3390/s25102967

**Published:** 2025-05-08

**Authors:** Yong Chang, Tengfei Gao, Juanhua Yang, Zongyao Liu, Biao Wang

**Affiliations:** 1 Henan Key Laboratory of Super Hard Abrasive Grinding Equipment, School of Mechanical and Electrical Engineering, Henan University of Technology, Zhengzhou 450001, China; tfeigao@163.com (T.G.); yjuanhua0528@163.com (J.Y.); liuzongyao@haut.edu.cn (Z.L.); 2 State Key Laboratory of Advanced Rail Autonomous Operation, Beijing Jiaotong University, Beijing 100044, China; wbiao@bjtu.edu.cn

**Keywords:** fault diagnosis, train transmission system, residual networks (ResNets), self-attention mechanism

## Abstract

The data-driven intelligent fault diagnosis method has shown great potential in improving the safety and reliability of train operation. However, the noise interference and multi-scale signal characteristics generated by the train transmission system under non-stationary conditions make it difficult for the network model to effectively learn fault features, resulting in a decrease in the accuracy and robustness of the network. This results in the requirements of train fault diagnosis tasks not being met. Therefore, a novel parallel multi-scale attention residual neural network (PMA-ResNet) for a train transmission system is proposed in this paper. Firstly, multi-scale learning modules (MLMods) with different structures and convolutional kernel sizes are designed by combining a residual neural network (ResNet) and an Inception network, which can automatically learn multi-scale fault information from vibration signals. Secondly, a parallel network structure is constructed to improve the generalization ability of the proposed network model for the entire train transmission system. Finally, by using a self-attention mechanism to assign different weight values to the relative importance of different feature information, the learned fault features are further integrated and enhanced. In the experimental section, a train transmission system fault simulation platform is constructed, and experiments are carried out on train transmission systems with different faults under non-stationary conditions to verify the effectiveness of the proposed network. The experimental results and comparisons with five state-of-the-art methods demonstrate that the proposed PMA-ResNet can diagnose 19 different faults with greater accuracy.

## 1. Introduction

With the advantages of large capacity, wide coverage, high cost-effectiveness, high safety, and good stability, railway transportation has always been an indispensable part of the transportation systems of countries around the world [1,2]. The safety and reliability of train operation are the basic guarantees for railway transportation safety; therefore, the fault diagnosis of key components of trains has always been a research focus for scholars. The train transmission system is the core component of the train power supply system, and its function is to transfer energy from electric machinery to wheelsets. Any unexpected failures of any component in the train transmission system may lead to serious safety accidents, resulting in unexpected casualties and significant economic losses. Therefore, developing effective fault diagnosis methods for reliability analysis and evaluation of train transmission systems and their key components has become an urgent problem to ensure the safe operation of trains [3,4].

Benefiting from the rapid development of advanced sensor technology and artificial intelligence algorithms, data-driven intelligent fault diagnosis methods have become effective and powerful tools for improving the safety and reliability of train operations [5,6,7,8,9]. Some promising results have been achieved with data-driven intelligent fault diagnosis methods for the train transmission system. Wang et al. [10] proposed a multi-attention one-dimensional convolutional neural network (MDCNN) for the diagnosis of wheel set faults by integrating the attention mechanism. Peng et al. [11] proposed a novel deep one-dimensional convolutional neural network (Der-1DCNN) for the diagnosis of wheel set bearing faults under load, variable speeds, and strong ambient noise disturbances, and achieved a high diagnostic accuracy under a strong noise environment. Wang et al. [12] proposed a novel multilayer wavelet integrated convolutional neural network (MWI-Net) for predicting wheel wear in high-speed trains and accurately predicted the wheel wear curve. Ding et al. [13] also proposed an evolvable fault diagnosis framework for the train driveline system by combining the system-level fault diagnostic network with an evolutionary learning mechanism and achieved a joint diagnosis of the whole system.

However, for the train transmission system, there are two challenging problems that seriously limit the application of intelligent fault diagnosis methods in practical engineering. The first challenge is the noise corruption in vibration signals, which is ubiquitous in mechanical fault diagnosis. Trains usually operate under complicated working conditions such as high speeds, heavy loads, and uneven rail surfaces. Vibration signals collected by sensors not only contain useful fault information but also strong environmental noise. Furthermore, other vibration information generated during the operation of other components in the train system will also be reflected in the measured signals. This noise and irrelevant information mask the representative fault features, and the useful fault information can hardly be extracted from the collected signals as a result [14,15]. The second challenge is the multi-scale characteristics of fault features, which means various local patterns with different lengths of data segments. Under nonstationary working conditions, the speed and load of the train during operation will change dramatically. The fault impulsive response of the transmission system is a time-varying signal, and the feature frequencies caused by faults vary at different scales. Moreover, there are multiple components in the whole transmission system, and each component has various types of faults. The vibration signals generated by different components and faults will be coupled with each other. Ultimately, the collected vibration signals contain complex feature information across multiple time scales and exhibit multi-scale characteristics in the frequency domain. The multi-scale characteristic leads to a sharp decline in the feature extraction capability and robustness of network models [16,17].

For the first challenge, the attention mechanism has been proven to be an effective technique to enhance the ability of other models to process monitoring signals. The attention mechanism accomplishes adaptive denoising by learning the relative importance degrees of relevant and irrelevant information in different feature maps. For the irrelevant information, the vibration response of the mechanical system and noise, which is unrelated to the fault diagnosis task, will be assigned lower weights, and the information related to the fault diagnosis task is given larger weights. Research has shown that the attention mechanism has become an important technique in the field of mechanical fault diagnosis [18,19,20]. For the second challenge, the multi-scale convolution model shows great potential. In the multi-scale convolution model, a multi-scale network structure is constructed by multiple convolutional layers with different convolution kernel sizes. Then, the features at different time scales and frequencies can be extracted automatically from vibration signals. Consequently, the performance and robustness of the deep network model are enhanced [21].

Inspired by the success of attention mechanisms and multi-scale convolutional models, researchers have proposed a variety of multi-scale networks incorporating attention mechanisms for the fault diagnosis of different mechanical systems [22,23]. For rolling bear fault diagnosis, the channel attention mechanism was introduced by Huang et al. [24] into a multi-scale CNN network to enhance the diagnosis performance. The fault features of the bearing were extracted by the maximum and average pooling layers from multiple scales, and the convolutional layer’s feature-learning ability was increased by the channel attention mechanism. In [25], a novel multiscale residual attention network model for bearing fault diagnosis was developed. In the proposed scheme, fault features at different scales were learned by an improved multiscale learning module, and a new residual attention learning strategy was developed to find the most informative features. Kim et al. [26] presented a multi-scale path attention residual network to improve the feature representation ability of multi-scale structures for rotating machinery fault features. The important multi-scale fault features could be effectively extracted by three multi-scale attention residual blocks, and then the extracted features were fused into the next part of the network.

Although multi-scale networks combined with attention mechanisms have effectively improved the application effectiveness of intelligent models on various research objects, for the train transmission system, there are still two obvious shortcomings in the existing intelligent network models that need to be addressed. (1) The generalization ability of the network model is insufficient to meet the fault diagnosis task requirements of the entire train transmission system. At present, the research objects of network models mainly focus on the key components in mechanical systems, such as bearings, gears, rotating machinery, etc. The train transmission system is a comprehensive mechanical structure composed of multiple components such as traction motors, wheelsets, transmission gears, and axle boxes. The types of faults and diagnostic requirements for different components vary greatly. The existing network models for individual components cannot meet the diagnostic requirements of the transmission system. (2) Under non-stationary operating conditions, the robustness and accuracy of the network model need to be further improved. The existing attention methods have insufficient ability to extract relevant information within the sequence and at each position, resulting in a decrease in fault diagnosis accuracy and feature richness of multi-scale network models. In addition, traditional attention methods such as channel attention and spatial attention are implemented in a cascading manner, which makes the model design more complex.

To overcome the above shortcomings, a novel parallel multi-scale attention residual neural network (PMA-ResNet) for a train transmission system is proposed in this paper. The main contributions of this work can be summarized as follows.

(1) Three multi-scale learning modules (MLMods) with different structures and convolutional kernel sizes are constructed based on the Inception network and residual neural network (ResNet), which improve the representation ability and robustness of the captured fault features even under non-stationary conditions. The multi-scale fault features of different components are effectively learned from the vibration signal at different scales.
(2) A parallel network structure is designed to improve the generalization ability of the proposed method, thereby meeting the fault diagnosis task requirements of the entire train transmission system.
(3) The self-attention mechanism strategy is introduced to improve the performance of the neural network by adaptively learning the relative importance of different feature maps and increasing the weights of useful features. Then, the learned features are further enhanced, providing rich and reliable feature information for the final classification results of the network model.
(4) A multi-operating condition, multi-component, and strong noise interference train transmission system fault simulation platform has been constructed, covering nine non-steady state operating conditions and 16 typical fault types. In comparison with the extant literature, which focuses on a single component or steady state condition, this experiment is more aligned with the actual train-operating environment.

The rest of this paper is structured as follows. Section 2 introduces the theoretical background of residual neural networks and inception networks. Then, the method of this paper is elaborated in Section 3. Experiments are conducted in Section 4 to evaluate the proposed method. Finally, conclusions are discussed in Section 5.

## 2. Theoretical Background

### 2.1. Residual Neural Network

The residual neural network (ResNet) [27] represents a major breakthrough in deep learning. The fundamental concept is the introduction of residual connectivity, which enables the network to concentrate on learning residual information in a deep network rather than learning constant transformations directly. This architectural approach permits the network to enhance its performance by increasing its depth, while circumventing the issue of performance deterioration associated with a network that is excessively deep.

The classical ResNet model consists of a convolutional layer, pooling layer, and fully connected (FC) layer.

(1) Convolutional Layer: The primary function of the convolutional layer is to process the input data by employing a convolutional kernel to extract local features from it. The convolution operation involves the sliding (or convolving) of the convolution kernel at each position of the input data. The resulting dot product of the filter and the input data at each position is then calculated, thereby generating the feature map. The following equation illustrates the convolution operation:

(1) $$x_{j}^{l}=\sigma (\sum_{i}k_{j,i}^{l}*x_{i}^{l-1}+b_{j}^{l})$$

where $x_{j}^{l}$ is the feature vector of the *j*th channel output from the *l*th layer; $\sigma \left(\cdot \right)$ is the activation function; $ck_{j,i}^{l}$ is the convolution vector of the *j*th channel in the *i*th convolution kernel; $x_{i}^{l-1}$ is the feature vector of the *l*th channel input from the *i*th layer; and $b_{j}^{l}$ is the bias vector of the convolution layer. After convolution, batch normalization and non-linearization of the activation function is required. In this paper, the ReLU activation function is selected and the expression is presented in (2).

(2) f(x)=max(0,x)

(2) Pooling layer: The pooling layer serves to reduce the dimension of the convolutional layer features, shorten the computation time, and inhibit the occurrence of the overfitting phenomenon. The pooling operation can be considered a statistical information extraction process. In this study, maximum pooling is employed, and the pooling operation can be expressed as follows:

(3) $$v_{j}^{l}=down(x_{j}^{l-1},s)$$

where $down\left(\cdot \right)$ is the maximum pooling down sampling function; $x_{j}^{l-1}$ is the feature vector of the *j*th channel extracted in the *l* − 1th convolutional layer; $v_{j}^{l}$ is the pooled feature vector; and *s* is the pooling parameter, which determines the dimensionality of the pooled features.

(3) Fully Connected Layer: The fully connected layer employs the features extracted from the preceding layers for the purpose of classification. It is structured as a multi-hidden layer artificial neural network (ANN), with the final layer comprising neurons that output the classification results. The output of the initial intermediate hidden layer can be expressed as follows:

(4) $$x^{l}=\sigma (\omega^{l}x^{l-1}+b^{l})$$

where $\omega^{l}$ is the weight matrix of the *l*th intermediate hidden layer and $b^{l}$ is the bias vector of the *l*th intermediate hidden layer.

### 2.2. Inception Network

The Inception network [28,29,30] is a deep convolutional neural network architecture proposed by Google in 2014, which has achieved notable results in a number of computer vision tasks. In contrast to traditional CNNs, which merely stack additional convolutional layers to augment the network’s depth, the Inception network addresses some of the pivotal challenges inherent to deep networks through the introduction of a parallel convolution strategy.

**Figure 1:**
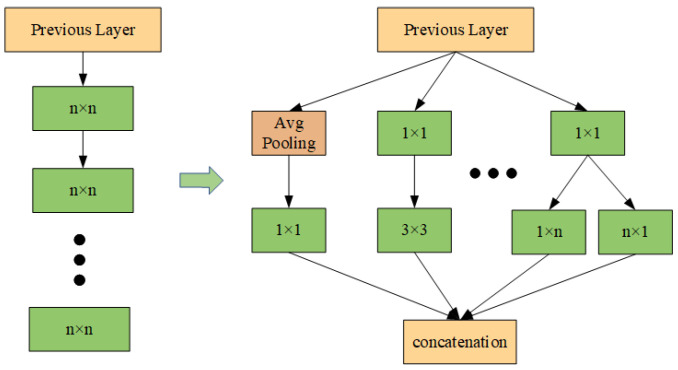
Inception network design ideas.

Prior to the inception of the Inception network, numerous popular CNN architectures attempted to enhance the performance of the model by increasing the number of convolutional layers. However, this approach presents a number of challenges. (1) A network that is excessively deep is prone to overfitting, and gradient propagation becomes increasingly difficult; (2) merely stacking larger convolutional layers increases computational costs. To address these issues, the Inception module introduces a parallel convolution strategy, whereby multiple convolutional kernels of varying sizes are applied to the same layer, thus processing the input data in parallel. The structure of the network becomes ‘wider’ rather than ‘deeper’, thereby enabling features to be extracted at different levels from multiple scales, as illustrated in Figure 1.

The Inception module is capable of capturing multi-scale information in the input data with greater comprehensiveness through the utilization of diverse scales of convolutional kernels, including 1 × 1, 3 × 3, 5 × 5, and so forth. This not only enhances the richness of feature expression but also ensures the network’s performance while markedly reducing the network depth and the number of parameters, thereby enhancing computational efficiency.

## 3. Proposed Method

This section provides a detailed description of the proposed PMA-ResNet method, including the multi-scale learning module, the self-attention mechanism, and the parallel structure.

### 3.1. Multi-Scale Learning Modules

**Figure 2:**
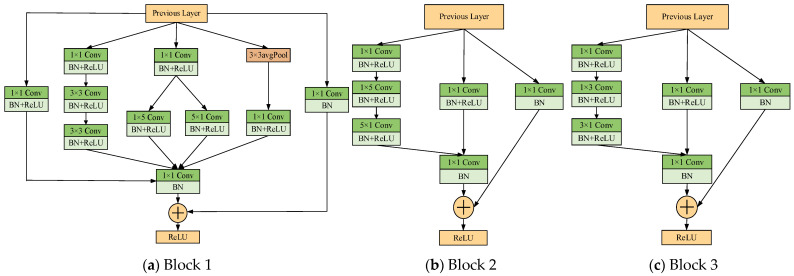
Multi-scale learning modules.

Inspired by the Inception network, multi-scale learning modules (MLMods) are designed in this paper. The designed MLMods are the layered structure comprising multiple convolutional layers and batch normalization (BN) layers, as illustrated in Figure 2. Among the aforementioned modules, the 1×1 convolution reduces the dimensionality of the input channel and subsequently performs the convolution operation, thereby effectively enhancing the network’s width. The decomposition of the n×n convolution kernel into two convolutions, 1×n and n×1, has the effect of reducing both the time and space complexities. Furthermore, the incorporation of a residual connection mitigates the issue of gradient vanishing due to network depth and enhances the overall generalization capability.

The convolutional layer of each module consists of multiple trainable convolutional kernels for extracting features of the input vibration signal, denoted as

(5) $$x^{l(i)}=\sigma (\sum K^{l(i)}*x^{l-1}+b^{l(i)})$$

where $x_{j}^{l\left(i\right)}$ is the output of the *l*th layer, $\sigma \left(\cdot \right)$ denotes the activation function, $K^{l\left(i\right)}$ denotes the weight of the *i*th convolutional kernel of the *l*th layer, $x^{l-1}$ is the input value of the *l*th layer, and $b^{l\left(i\right)}$ is the bias vector of the convolutional layer.

In order to obtain features at different scales and ensure the quality of these features, it is first necessary to extract the signal features through a multi-scale convolutional layer. These features are then fused.

(6) $$\begin{array}{l} x^{l(i,j)}=\sigma (\sum K^{l(i,j)}*x^{l(i)}+b^{l(i,j)})\\ Z^{l(i)}=Concat(x^{l(i,1)},\;x^{l(i,2)},\;x^{l(i,3)},\;x^{l(i,4)}) \end{array}$$

Stacking convolutional layers can deepen the network structure, but an increase in the number of layers may cause the gradient to disappear during backpropagation. This problem can be solved effectively by introducing BN layers. The BN process is expressed as

(7) $$\begin{array}{l} \mu =\frac{1}{N_{batch}}\sum_{i=1}^{N_{batch}}x_{i}\\ \rho^{2}=\frac{1}{N_{batch}}\sum_{i=1}^{N_{batch}}(x_{i}-\mu {)}^{2}\\ {\bar{x}}_{i}=\frac{x_{i}-\mu }{\sqrt{\rho^{2}+\varepsilon }}\\ BN_{\gamma ,\beta }(x_{i})=\gamma {\bar{x}}_{i}+\beta \end{array}$$

where $N_{batch}$ is the batch size, $x_{i}$ is the input element per batch, μ is the batch mean, $\rho^{2}$ is the batch variance, and ${\bar{x}}_{i}\;$ is the normalized value.

### 3.2. Self-Attention Mechanism

It is often the case that traditional convolutional neural networks fail to fully appreciate the significance and relevance of different features when engaged in the process of feature extraction. Furthermore, the constraints imposed by limited computational resources serve to restrict the extent of their processing capabilities. The incorporation of an attention mechanism into the network facilitates the prioritization of salient information within the input data, thereby enhancing the model’s accuracy and computational efficiency.

**Figure 3:**
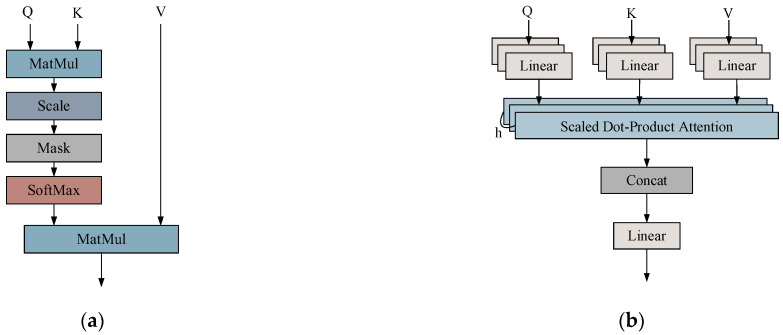
Scaled dot product attention (**a**); multiple attention mechanisms (**b**).

The self-attention mechanism [31] is a process that enables the dynamic adjustment of the representation of each element by calculating the similarity between elements in the input sequence. This mechanism facilitates the capture of global dependencies by assigning a weight to each element based on its relationship with other elements during the computation of its representation. As illustrated in Figure 3a, scaled dot product attention (SDPA) is demonstrated. The fundamental aspect of the methodology is the calculation of the correlation between each element in the input sequence and other elements, which can be conceptualized as attention weights. These weights are then employed to weight and sum the sequence, thereby obtaining a new representation. This representation not only preserves the information of all elements in the sequence but also identifies the salient portions relevant to the current element.

The computational process of the self-attention mechanism consists of the following steps:

(1) Vector representation: For an input vector of *X*, each element in the sequence generates the corresponding query vector *Q*, key vector *K*, and value vector *V*.

(8) $$Q=W^{Q}\cdot X,K=W^{K}\cdot X,V=W^{V}\cdot X$$

where $W^{Q}$, $W^{K}$, and $W^{V}$ are learnable weight matrices.

(2) Calculate relevance: For each query vector, calculate its similarity with all key vectors by the dot product to get the attention score.

(9) $$Attention\;Score(i,j)=Q_{i}\cdot K_{j}^{T}$$

To prevent the inner product value from becoming excessively large due to high dimensions, which can lead to vanishing or exploding gradients in the SoftMax function, the dot product result is typically scaled by a factor proportional to the dimensions of *Q* and *K*. This scaling factor helps control the magnitude of the inner product.

(10) $$Scaled\;Attention\;Score(i,j)=\frac{Q_{i}\cdot K_{j}^{T}}{\sqrt{d_{k}}}$$

(3) Normalization: Weights: The attention scores are converted to attention weights by the SoftMax function such that they sum to one.

(11) $$\alpha_{ij}=Soft\;\max\left(\frac{Q_{i}\cdot K_{j}^{T}}{\sqrt{d_{k}}}\right)$$

(4) Compute the attentional output: The attentional output is a weighted sum of the value vectors.

(12) $$Attention\;Output(i)=\sum_{j}\alpha_{ij}\cdot V_{j}$$

In contrast, the multi-head attention mechanism is founded upon the dot product attention mechanism, which employs a series of linear transformations to partition the input vector into multiple subspaces (i.e., ‘heads’). Each subspace then undergoes a calculation of the dot product attention independently, after which the outputs of the various subspaces are spliced together and subjected to a linear transformation. This design enables the model to concurrently attend to disparate information within distinct representation subspaces, thereby augmenting the model’s expressiveness and robustness. The structure of the multi-attention mechanism is illustrated in Figure 3b.

The formula for the operation of the multi-attention mechanism with input *X* is as follows:

(13) $$MultiHead(X,h,W^{Q},W^{K},W^{V},W^{O})=concat(head_{1},\cdots ,head_{h})W^{O}$$

where *h* is the number of heads and $W^{O}$ is the output projection matrix.

Each weight matrix is formed by concatenating the weight matrices of each header. The output of the result of each representation header operation is given by the following equation:

(14) $$head_{i}=selfAttention(XW_{i}^{Q},XW_{i}^{K},XW_{i}^{V})$$

### 3.3. Parallel Network Structure

The traditional serial network structure relies on layer-by-stacked network layers for information transfer, but may face the following challenges during deep network training: (1) as the network depth increases, the gradient may gradually weaken during backpropagation, which affects the training effect of the model; (2) the computation of the serial structure relies on the outputs of the previous layer, which makes it difficult to make full use of the parallel computing capability of modern computing architectures; and (3) during the inter-layer transfer process, some local features may be lost, making the model limited in capturing global information. In contrast, the parallel structure can process multiple feature streams simultaneously, improve data throughput, reduce information loss, and alleviate the gradient-vanishing problem to a certain extent, thus improving the stability and computational efficiency of the deep network.

**Figure 4:**
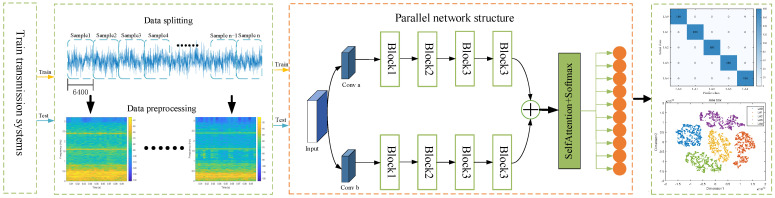
The overall architecture of the proposed PMA-ResNet.

According to Ensemble Learning Theory, the collaborative work of multiple learners usually possesses stronger generalization ability than a single learner. The parallel network structure proposed in this paper (as in Figure 4, the parallel network structure part) can be regarded as a special form of ensemble learning, which enhances the model’s ability to learn different feature dimensions through multi-branch feature extraction and improves the overall generalization ability through the fusion strategy.

The introduction of parallel structure mainly optimizes the model performance in the following aspects:

(1) Feature diversity: Despite the same network structure in both branches, the parallel structure is able to learn different feature distributions due to the randomness of parameter initialization and differences in data flow, thus improving feature representation.
(2) Information redundancy: Feature extraction from multiple independent paths enables the model to characterize the data from different levels and perspectives, which helps to reduce the risk of underfitting or overfitting that a single path may bring.
(3) Feature fusion: In the final fusion stage, the features extracted from different paths are complemented and enhanced in the high-dimensional space, so as to obtain a more discriminative feature representation and improve the fault diagnosis performance of the model.

### 3.4. PMA-ResNet Overall Structure

This paper introduces the multi-scale learning module and self-attention mechanism into the parallel network, leading to the proposal of the PMA-ResNet model for the diagnosis of faults in rotating machinery. The overall architecture of the proposed PMA-ResNet is illustrated in Figure 4, and the parameter settings of the network are presented in Table 1.

The steps involved in the diagnosis of faults using the PMA-ResNet method are as follows.

(1) Data preprocessing: The input data are intercepted for initial preprocessing through an equal-length sliding window, a process that can be referred to as data augmentation. Subsequently, the data are normalized to a specific range in order to ensure consistency, and the corresponding time–frequency maps are generated. These are then divided into a training set and a test set.

(2) Model training: The training set is employed as an input to train the network, and feature extraction is conducted by the multi-scale learning module, which acquires features from the attention layer and the fully connected layer. The Adam algorithm is utilized to update the network parameters by back propagation.

(3) Fault diagnosis: The input signals are introduced into a trained PMA-ResNet model, and the ultimate health of the part is determined by a classifier.

## 4. Experimental Validation

In this section, the focus is on conducting fault experiments on the train drive train, with the objective of evaluating the proposed PMA-ResNet method using experimental data, in comparison with five alternative methods. The five methods under consideration are LeNet-5 [32], AlexNet [33], VGG-16 [34], ResNet-18 [27], and Inception-v3 [29]. Subsequent to this, ablation experiments are designed to facilitate a more profound comprehension of the contribution of each component of the proposed model.

### 4.1. Experimental Scheme

**Figure 5:**
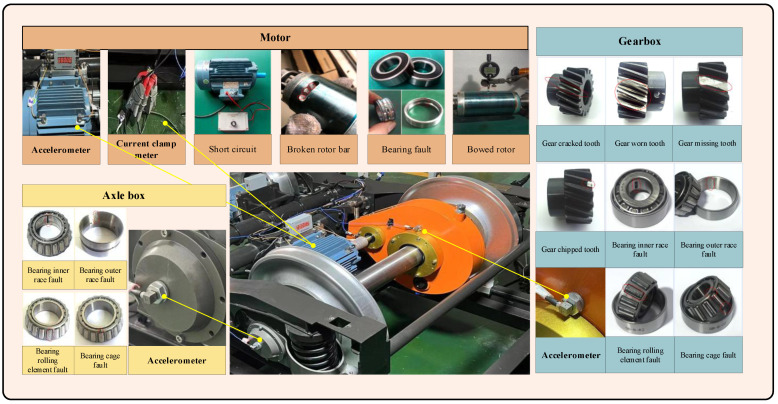
Experimental platform and typical fault parts.

In order to ascertain the efficacy of the proposed network, this paper presents the findings of a fault simulation experiment conducted on the metro train drive train system. The experiment involved the collection of multi-source sensor signals under a range of health states. The experimental platform is an urban bogie failure simulation experimental bench, as illustrated in Figure 5. The ratio of the experimental platform to the real bogie is 1:2. As illustrated in Table 2, the dataset encompasses nine distinct operational scenarios. The different speeds are employed to simulate varying train speeds, while the different lateral loads are used to simulate straight lines or rounded corners. The experiment involved the collection of two types of signals: three-way vibration and three-phase current. Two triaxial acceleration sensors were positioned at the driving and non-driving ends of the motor, while a three-phase ammeter was placed at the motor cable. A triaxial acceleration sensor was installed at the input and output shafts of the gearbox, as well as at the axial endcap, resulting in a total of 18 channels. The sampling frequency of each channel was set to 64 kHz. The experimental data were collected using a DAQ data collector. The experiment simulates the effects of different health states of the train drive train components (motors, gearbox, and axle box bearings), including normal and 16 typical single faults. Images of the faulty components of the motor, gearbox, and axle box are shown in Figure 5.

In the data preprocessing stage, the signal is segmented by fixed-length sliding windows, each of which has a length of 6400 sample points (i.e., 0.1 s), and a step size of 6400 points as well, with a non-overlapping window to avoid redundancy and to ensure the diversity of the samples. In order to extract the time–frequency characteristics of the signal, the short-time Fourier transform (STFT) was used to convert the one-dimensional signal into a two-dimensional time–frequency map (e.g., Figure 4, data preprocessing section). The parameters are set to Hamming window (window length 2048, overlap 1024, FFT points 2048), and this configuration can effectively balance the time and frequency resolution. Finally, all time–frequency images were normalized in the range of [0, 1] to ensure training stability and accelerate convergence.

### 4.2. Implementation Details and Evaluation Metrics

The algorithm proposed in this paper has been implemented in MATLAB R2023a, running on an Intel Xeon E5-2687W CPU with 128 GB of RAM (HP Inc., product of Beijing, China). During training, the cross-entropy loss function and the Adam optimization algorithm are used, with a learning rate of 0.0001 and a batch size of 128. In the subsequent experiments, accuracy is employed as the primary metric to evaluate the algorithm’s performance. Accuracy is defined as follows:

(15) $$A=\frac{N^{c}}{N}$$

where *N* is the total number of classified objects, $N^{c}\;$ is the total number of correctly classified objects, and *A* is the accuracy.

### 4.3. Experiment on Axle Box Dataset

The bearing dataset represents a typical fault dataset that is frequently employed as a benchmark for the assessment of algorithms. In this section, the performance of the algorithm is evaluated using the axle box data, which contain five common faults of bearings: normal (LA0), bearing inner race fault (LA1), bearing outer race fault (LA2), bearing rolling element fault (LA3), and bearing cage fault (LA4), as shown in Table 3. To facilitate a more comprehensive evaluation of the proposed algorithms and facilitate comparison with other algorithms, the training sample size was increased from 5% to 80%. The diagnostic accuracy of each algorithm on axle box data is presented in Table 4.

As can be observed in Table 4, the PMA-ResNet algorithm demonstrates superior performance compared to the other algorithms under all conditions. With regard to the size of the training sample, PMA-ResNet attains an accuracy of 85.89% with a training sample size of 5%, which is approximately 18% higher than that of the second-best algorithm, ResNet-18. In contrast, other conventional convolutional neural networks, such as VGG-16 and LeNet5, are prone to overfitting at lower training sample sizes. Consequently, the accuracy of these algorithms declines significantly when the training sample size is reduced. Nevertheless, PMA-ResNet continues to demonstrate superior accuracy with reduced training sizes. PMA-ResNet exhibits enhanced feature extraction capabilities, as evidenced by its superior performance compared to other algorithms across diverse training sample sizes.

**Figure 6:**
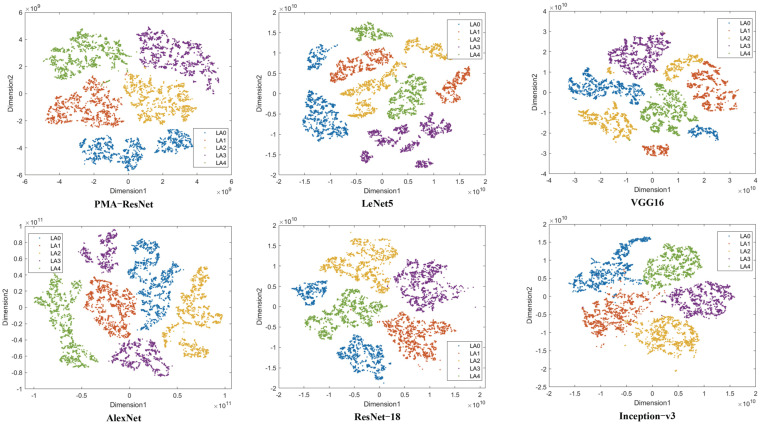
Feature visualization on axle box dataset.

In order to facilitate a more comprehensive analysis of the feature extraction capability, the features extracted by the aforementioned six algorithms were projected into a two-dimensional space (utilizing the default parameter settings) through the implementation of the t-SNE visualization technique. The resulting data are presented in Figure 6. Figure 6 demonstrates the superiority of PMA-ResNet over alternative methodologies in differentiating between disparate classes of samples. In particular, PMA-ResNet is effective in clustering data points into distinct classes with high similarity within each class and significant differences between classes, thereby enhancing classification performance.

### 4.4. Experiment on Gearbox Dataset

Gearboxes are integral components of industrial machinery, typically comprising gear sets and support bearings. The dataset comprises information on common gear and support bearing failures. These include normal (G0), gear cracked tooth (G1), gear worn tooth (G2), gear missing tooth (G3), gear chipped tooth (G4), bearing inner race fault (G5), bearing outer race fault (G6), bearing rolling element fault (G7), and bearing cage fault (G8), as shown in Table 5. As with the shaft box experiments, the performance of the different methods is initially evaluated at varying sample sizes. The training sample sizes are also taken to be 5%, 10%, 30%, 50%, and 80%. The classification accuracies are initially presented in Table 6 in order to demonstrate the direct performance on the gearbox data.

As illustrated in Table 6, PMA-ResNet exhibits robust performance across a range of training sample sizes. In particular, when the training sample size is relatively small, for example, 5% or 10%, PMA-ResNet still achieves diagnostic accuracies of 92.30% and 98.15%. This result is approximately 24% and 20% higher than that of the second method. Furthermore, as the number of training samples increases, PMA-ResNet achieves near-perfect accuracy, which remains slightly higher than that of the other compared CNNs. In other words, PMA-ResNet is not only capable of accurately extracting features in the presence of limited training data, but it also outperforms traditional CNNs in scenarios where a large training set is available.

**Figure 7:**
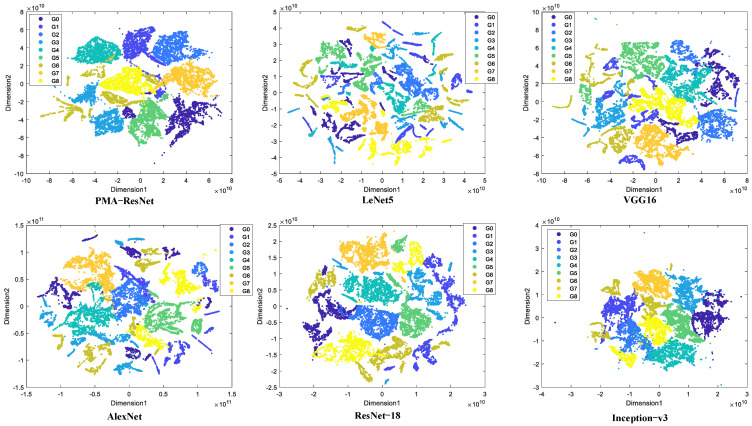
Feature visualization on gearbox dataset.

As illustrated in Figure 7, PMA-ResNet effectively categorizes diverse sample types into their corresponding regions. While a minor proportion of misclassifications exists, the overall classification accuracy is demonstrably high. In the case of other convolutional neural networks, such as VGG-16 and AlexNet, the presence of a multitude of fault features makes it challenging to differentiate between them, resulting in a lower classification accuracy.

### 4.5. Experiment on Motor Dataset

As shown in Table 7, the motor data are represented by five states: normal, short-circuit, broken rotor bar, bearing failure, and bowed rotor. These states are labelled M0, M1, M2, M3, and M4, respectively. The dataset comprises both vibration and current signal types. The same experiments were conducted on this dataset as previously described in order to evaluate the performance of the model in diagnosing motor faults.

**Figure 8:**
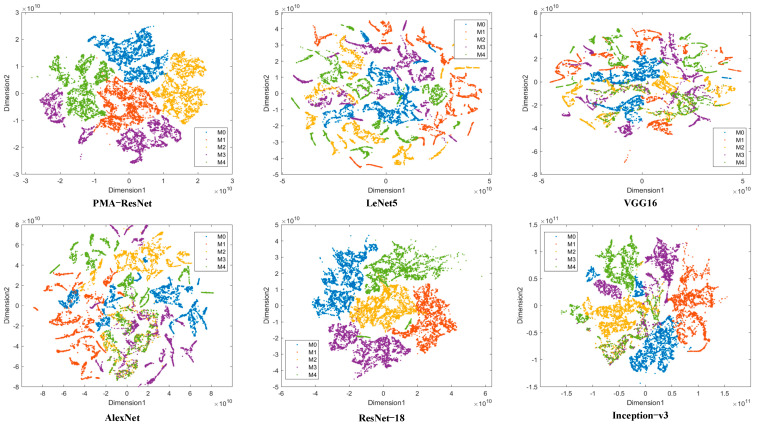
Feature visualization on motor dataset.

A comparison of Table 8 and Figure 8 reveals that PMA-ResNet continues to demonstrate superior performance relative to other algorithms. Notably, PMA-ResNet demonstrates considerable efficacy with a limited training sample size. For instance, it attains an accuracy of 89.49% with merely 5% of the training samples. Therefore, it can be concluded that PMA-ResNet performs well with small samples. Furthermore, PMA-ResNet demonstrates efficacy even when utilizing typical training set sizes, attaining 100% accuracy with 50% and 80% of the training samples. With regard to the classification of faults, the conventional convolutional neural networks (CNNs) such as LeNet, AlexNet, and VGG-16 are unable to effectively extract features due to the coexistence of two signal types and a multitude of fault characteristics, which ultimately leads to a decline in classification accuracy. In comparison, PMA-ResNet demonstrates the capacity to accurately identify all types of samples, with only a minimal number of misclassifications.

### 4.6. Ablation Experiments

In order to gain further insight into the function of each component of the proposed model, three pertinent ablation experiments are designed in the following section. The methodology employed in these experiments is outlined below:

(1) Method A: a traditional residual neural network is used as the training model;
(2) Method B: introducing a multi-scale learning module on the basis of Method A;
(3) Method C: introducing a self-attention mechanism on the basis of Method B;
(4) Method D: a parallel network structure, the PMA-ResNet diagnostic model, is introduced on the basis of Method C.

In this experiment, the aforementioned data pertaining to motor, gearbox, and axle box failures were selected for analysis on a standalone basis. Furthermore, 80% of the samples were randomly selected for training purposes, while the remaining 20% were designated for testing. In order to mitigate the impact of random variation, each set of experiments was conducted 10 times.

**Figure 9:**
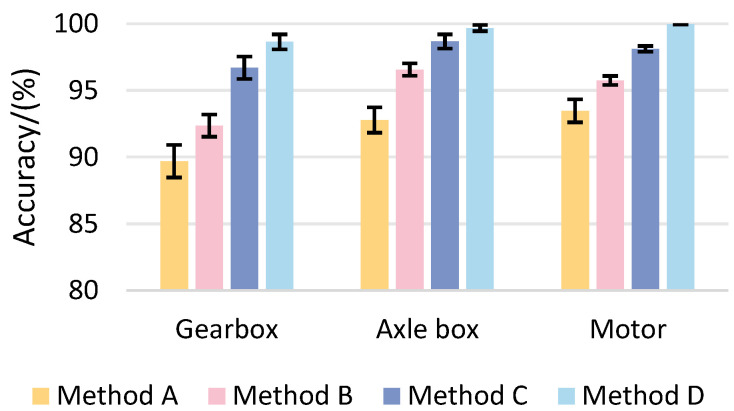
Comparison of the diagnostic accuracy of each method of ablation test.

The results of the ablation experiments are shown in Table 9 and Figure 9. The results of the ablation experiments demonstrate that the diagnostic accuracy of Method B is significantly enhanced in all three devices when compared to Method A. The average increase in accuracy is 2.67%, 3.78%, and 2.28%, respectively. This improvement substantiates the assertion that the incorporation of the multi-scale learning module markedly enhances the capture of fault feature information and fortifies the network’s capacity for feature extraction and classification. This exemplifies the efficacy of the multi-scale learning module. Method C, based on Method B, introduces the self-attention mechanism, resulting in further improvement in diagnostic accuracy, particularly in the case of the gearbox. This indicates that the self-attention mechanism has a significant impact on enhancing the model’s capacity to focus on intricate data. In comparison to Method C, the complete method, comprising a parallel structure, multi-scale learning module, and self-attention mechanism, exhibits exceptionally high diagnostic accuracy with regard to the fault data of the gearbox, axle box, and motor. This serves to validate the efficacy and superiority of the method.

## 5. Conclusions

This paper proposes a PMA-ResNet model for the diagnosis of train driveline faults. This model combines multiscale learning and a self-attention mechanism, and is able to extract multiscale features from input signals and distinguish important features effectively. Specifically, a multi-scale learning module was designed to extract multi-scale features through convolutional kernels of different sizes; meanwhile, the self-attention mechanism assigns weights to the features and performs weighted summation, which effectively reduces the noise interference and enhances the useful information to improve the diagnostic accuracy of the network. In addition, the network adopts a parallel structure to enhance its generalization ability. The experimental part constructs a train transmission system fault experimental bench, and the collected experimental data are closer to the actual working conditions. The effectiveness of the proposed method is verified by the collected data. The experimental results show that PMA-ResNet exceeds the other five existing methods in terms of feature extraction capability and diagnostic accuracy, thus confirming the superiority of the model. The ablation experiments further validate the superiority of each module. By introducing the multi-scale learning module, self-attention mechanism, and parallel network structure, the average diagnostic accuracies of the model for each component increase by 2.91%, 2.94%, and 1.6%, respectively, which verifies the superiority of each module in improving the diagnostic performance of the model.

## Figures and Tables

**Table 1:** Network parameters.

Network Module	Parameters	Output Dimensions
Input Layer	——	128 × 128 × 3
Conv1	(7,7,32)	128 × 128 × 32
Maxpooling layer	(5,5,2)	64 × 64 × 32
Block1	——	64 × 64 × 64
Maxpooling layer	(5,5,2)	32 × 32 × 64
Block2	——	32 × 32 × 96
Block3	——	32 × 32 × 128
GAP	(5,5,2)	16 × 16 × 128
Self-attention Layer	(4,64)	64 × 1
Fully Connected Layer	19 × 1	——
Output Layer	——	19

**Table 2:** Summary of working conditions.

Working Condition Number	Motor Speed/Load
WC1	20 Hz/0 kN
WC2	20 Hz/10 kN
WC3	20 Hz/−10 kN
WC4	40 Hz/0 kN
WC5	40 Hz/10 kN
WC6	40 Hz/−10 kN
WC7	60 Hz/0 kN
WC8	60 Hz/10 kN
WC9	60 Hz/−10 kN

**Table 3:** Health state in the axle box datasets.

Node	Label	Health State
Axle box	LA0	Normal condition
	LA1	Bearing inner race fault
	LA2	Bearing outer race fault
	LA3	Bearing rolling element fault
	LA4	Bearing cage fault

**Table 4:** Diagnostic accuracy of axle box data.

Method	Diagnostic Accuracy (Axle Box)				
	0.05	0.1	0.3	0.5	0.8
LeNet5	43.30%	61.33%	99.17%	99.67%	99.69%
VGG-16	27.67%	42.79%	72.03%	99.11%	99.78%
AlexNet	20.19%	48.64%	92.92%	99.78%	99.78%
ResNet-18	67.88%	91.19%	97.19%	99.16%	99.56%
Inception-v3	40.91%	54.94%	82.48%	98.04%	99.44%
PMA-ResNet	85.89%	98.79%	99.81%	1	1

**Table 5:** Health state in the gearbox datasets.

Node	Label	Health State
Gearbox	G0	Normal condition
	G1	Gear cracked tooth
	G2	Gear worn tooth
	G3	Gear missing tooth
	G4	Gear chipped tooth
	G5	Bearing inner race fault
	G6	Bearing outer race fault
	G7	Bearing rolling element fault
	G8	Bearing cage fault

**Table 6:** Diagnostic accuracy of gearbox data.

Method	Diagnostic accuracy (Gearbox)				
	0.05	0.1	0.3	0.5	0.8
LeNet5	42.06%	86.21%	93.07%	94.93%	97.53%
VGG-16	28.75%	37.26%	78.93%	97.90%	98.46%
AlexNet	37.47%	57.41%	92.40%	93.49%	94.44%
ResNet-18	68.49%	78.54%	93.89%	98.81%	99.90%
Inception-v3	42.94%	56.20%	86.31%	96.14%	99.81%
PMA-ResNet	92.30%	98.15%	98.77%	99.62%	99.97%

**Table 7:** Health state in the motor datasets.

Node	Label	Health State
Motor	M0	Normal condition
	M1	Short circuit
	M2	Broken rotor bar
	M3	Bearing fault
	M4	Bowed axis

**Table 8:** Diagnostic accuracy of motor data.

Method	Diagnostic Accuracy (Motor)				
	0.05	0.1	0.3	0.5	0.8
LeNet5	73.65%	79.56%	90.34%	91.19%	96.04%
VGG-16	24.45%	59.15%	85.99%	87.75%	90.33%
AlexNet	52.76%	66.12%	86.25%	89.70%	94.89%
ResNet-18	59.81%	75.25%	94.22%	99.94%	1
Inception-v3	76.81%	85.84%	89.17%	92.83%	92.78%
PMA-ResNet	89.49%	96.95%	99.98%	1	1

**Table 9:** Diagnostic accuracy of ablation experiments.

Method	Diagnostic Accuracy		
	Gearbox	Axle Box	Motor
Method A	89.69% ± 1.22%	92.78% ± 0.95%	93.46% ± 0.86%
Method B	92.36% ± 0.83%	96.56% ± 0.46%	95.74% ± 0.34%
Method C	96.69% ± 0.84%	98.67% ± 0.53%	98.11% ± 0.22%
Method D	98.64% ± 0.56%	99.67% ± 0.23%	99.96% ± 0.03%

## Data Availability

The original contributions presented in the study are included in the article; further inquiries can be directed to the corresponding author.
